# Can the early use of botulinum toxin in post stroke spasticity reduce contracture development? A randomised controlled trial

**DOI:** 10.1177/0269215520963855

**Published:** 2020-10-11

**Authors:** Cameron Lindsay, Sissi Ispoglou, Brinton Helliwell, Dawn Hicklin, Steve Sturman, Anand Pandyan

**Affiliations:** 1 School of Allied Health Professions, Keele University, Staffordshire, UK; 2 Ulster Hospital, South Eastern HSC Trust, Belfast; 3 Department of Elderly Medicine, Sandwell Hospital, West Bromwich, West Midlands, UK; 4 Neurology Department, Queen Elizabeth Hospital, Birmingham, UK

**Keywords:** Stroke, spasticity, botulinum toxin, arm, upper limb

## Abstract

**Objective:**

Does early treatment of spasticity with botulinum-toxin (BoNTA), in (hyper)acute stroke patients without arm-function, reduce contractures and improve function.

**Design:**

Randomised placebo-controlled-trial

**Setting:**

Specialised stroke-unit.

**Participants & Intervention:**

Patients with an Action Research Arm Test (ARAT) grasp-score⩽2 who developed spasticity within six-weeks of a first stroke were randomised to receive injections of: 0.9%sodium-chloride solution (placebo) or onabotulinumtoxin-A (treatment).

**Outcome-Measures:**

Spasticity, contractures, splint use and arm function (ARAT) were taken at baseline, 12-weeks post-injection and six-months after stroke. Additionally, spasticity and contractures were measured at weeks-two, four and six post-injection.

**Results:**

Ninety three patients were randomised. Mean time to intervention was 18-days (standard deviation = 9.3). Spasticity was lower in the treatment group with difference being significant between week-2 to 12 (elbow) and week-2 to 6 (wrist). Mean-difference (MD) varied between –8.5(95% CI –17 to 0) to –9.4(95% CI –14 to –5) µV.

Contracture formation was slower in the treatment group. Passive range of motion was higher in the treatment group and was significant at week-12 (elbow MD6.6 (95% CI –0.7 to –12.6)) and week-6 (wrist MD11.8 (95% CI 3.8 to 19.8)). The use of splints was lower in the treatment group odds ratio was 7.2 (95% CI 1.5 to 34.1) and 4.2 (95% CI 1.3 to 14.0) at week-12 and month-6 respectively.

Arm-function was not significantly different between the groups MD2.4 (95% CI –5.3 to 10.1) and 2.9 (95% CI –5.8 to 11.6) at week-12 and month-6 respectively.

**Conclusion:**

BoNTA reduced spasticity and contractures after stroke and effects lasted for approximately 12-weeks. BoNTA reduced the need for concomitant contracture treatment and did not interfere with recovery of arm function.

**Trial Registration:**

EudraCT (2010-021257-39) and ClinicalTrials.gov-Identifier: NCT01882556.

## Introduction

Recovery of arm function in people who survive a stroke is commensurate with severity of impairment at stroke onset.^1^ Those people who have severe impairment of the arm at onset and who do not recover useful arm function, are likely to develop contractures.^2,3^ Contracture formation is exacerbated in those who have certain forms of spasticity.^4–6^ It can therefore be hypothesised that the lack of movement (as a result of the paralysis) in addition to the fixed positioning (associated with some forms of spasticity) accelerate the formation of contractures.^6^

Contractures are characterised by the combination of increased stiffness and loss of range of movement at a joint. Contractures can be established within four weeks of a stroke and 52% of stroke survivors have developed a contracture at six months.^4,7,8^ In some cases where motor recovery is delayed, it is possible that contractures could limit the recovery of meaningful function rather than the lack of neuro-plastic potential.

One way of slowing contracture development is through intensive mobilisation using cyclical electrical stimulation and this has been demonstrated previously in stroke patients at risk of wrist flexion contractures.^9^ The aim of this study was to explore if preventing the fixed positioning associated with spasticity (using additional treatment with botulinum toxin) could reduce both contractures and the rate at which contractures were formed. It was hypothesised that the reduction of spasticity and contractures would lead to a subsequent improvement in arm function. This study, therefore, also aimed to quantify changes in arm function following treatment.

## Methods

This phase II, double-blind, randomised, placebo-controlled, single-centred trial, with an initial screening phase was approved by North West - Greater Manchester South Ethics Committee Reference number 10/H1003/111. It was registered with EudraCT (2010-021257-39) and the trial protocol has been published.^10^ The Trial was registered at: ClinicalTrials.gov-Identifier: NCT01882556. The study protocol was developed in consultation with stroke patients one of these patients (BH) remained as a part of the trial steering committee.

Stroke patients admitted to a stroke unit in a tertiary care district general hospital were eligible to participate if they were; aged over 18, with a diagnosis of a first stroke within the last 42 days, and had a score of less than or equal to two on the easiest pick and place task on the grasp subsection of the Action Research Arm Test (ARAT) (i.e. lift and place a 2.5 cm^3^ wooden block).^11^ The exclusion criteria were; significant musculoskeletal conditions prior to stroke, contra-indications to electrical stimulation, known previous spasticity, hypersensitivity to excipients of BoNTA, infection at the proposed injection sites, or pregnancy.

Following written consent, either by the patient or their legal representative, screening for spasticity (described below) was carried out every Monday, Wednesday and Friday, by a physiotherapist, from recruitment for a period of six weeks from stroke onset.

If during this screening period, the patient

a. developed spasticity **and**
b. had a score of less than or equal to two on the grasp subsection of the Action Research Arm Test (ARAT)

they were randomised to either the treatment or control group.

Randomisation was done by computer-generated, random permuted blocks in a pseudorandom sequence. An independent research pharmacist assigned patients according to randomisation and dispensed the appropriate vials of 0.9% sodium chloride solution +/– Onabotulinumtoxin-A for two clinicians to reconstitute. The injecting clinician was therefore handed uniformly appearing syringes ensuring that the patient, injecting clinician and assessor were all blinded to the treatment being delivered.

Intramuscular injections of Onabotulinumtoxin-A (Allergan Ltd. Eire) (treatment group) or 0.9% sodium chloride solution (placebo group) to all six muscles of the affected arm in predetermined doses (Table 1). Localisation of the involved muscles was determined primarily by electrical stimulation techniques and where this was not possible by using ultrasound imaging.

**Table 1.** Muscles and dose of onabotulinumtoxin-A injected.

Muscle injected	Dose (Units)	Volume of saline for reconstitution (ml)
Biceps	40	0.8
Brachialis	40	0.8
Flexor digitorum superficialis	25	0.5
Flexor digitorum profundus	25	0.5
Flexor carpi ulnaris	15	0.3
Flexor carpi radialis	15	0.3

Electrical stimulation to the wrist extensors was provided to all patients recruited to the trial.^9^ Electrical stimulation was used to produce a movement through the full range of wrist extension while optimising participant comfort (pulse width was set to 300 μs; frequency was set to 40 Hz with an on time of 30 seconds including a five second ramp up and five second ramp down followed by a 30 seconds off time) for a period of ninety days.

The following outcome measures were taken by an independent assessor who was involved in recruitment and screening.

- Spasticity was measured neurophysiologically, using the muscle activity (EMG) response of a muscle to a passive stretch (Supplemental Appendix 1), and using the Tardieu Scale.^9,10,12,13^ The neurophysiological measure is a direct measure of spasticity whereas the Tardieu Scale is an indirect measure of spasticity.
- Contractures, at the wrist and elbow, were measured by quantifying passive range of extension and (b) stiffness encountered during the testing of passive range of extension.^9,10,12,13^ (Supplemental Appendix 1). The zero, at the wrist and elbow, corresponded with the anatomical neutral.
- The additional resources, for example, splints or casts, used to treat contracture were also documented.
- Arm function was measured using the Action Research Arm (ARAT).^11^

In the original protocol,^10^ arm function (measured by the ARAT) was identified as the primary measure, however, as the main aim of the intervention under investigation was to treat spasticity and the concomitant contractures these were identified as the main measures for this report.

Following a baseline measurement, repeated measurements were taken 12-weeks (three months) post injection and six months post stroke. Additional measures of spasticity and contractures were taken at two, four and six weeks after injections.

Those needing additional treatment for contractures and prescribed with splints were identified using clinical records at three months post injection and six months post stroke.

### Data analysis

Preliminary sample size calculations were conducted using the measure of arm function from a previous pilot study.^13^ With an effect size of 0.5 (using the ARAT) and at 80% power and a 0.05 significance level 126 patients would be required for a two arm RCT.

The pre-agreed intent-to-treat (ITT) population included all those patients who were randomised and received their injection of study medication at baseline.

Data was analysed using SPSS Statistics (v21, IBM, USA).

- The baseline and demographic data were presented with appropriate descriptive statistics (mean and standard deviations (SD)) or percentages.
- There were two methods used to measures of spasticity, a direct measure (muscle activity to a passive stretch) and an indirect measure (the Tardieu Scale). The performance of these two measures were compared to confirm congruence and sensitivity of the Tardieu Scale was calculated.
- The outcome measures for spasticity and contractures were reported at each measurement time point as the mean and SD. The mean difference and the 95% Confidence Interval (95% CI) are also reported.
- The rate at which contractures developed was calculated by quantifying the rate of change in passive range of extension (degrees/week) estimated from the repeated measures taken between baseline and 12-weeks post injection. The summary methods recommended by Matthews et al.,^14^ that is, the slope of the regression line using the least square estimate, was used to calculate the rate of change for the control and treatment group respectively. The differences in this rate of change is reported.
- A common physical treatment for developing contracture is the use of a splint. The odds ratio (OR) for needing treatment with a splint and 95% CI is reported.
- The men differences in arm function at three months post injection and six months post stroke along with the 95% CI. The respective p-values from an independent sample t-test is also reported.

## Results

**Figure 1.**
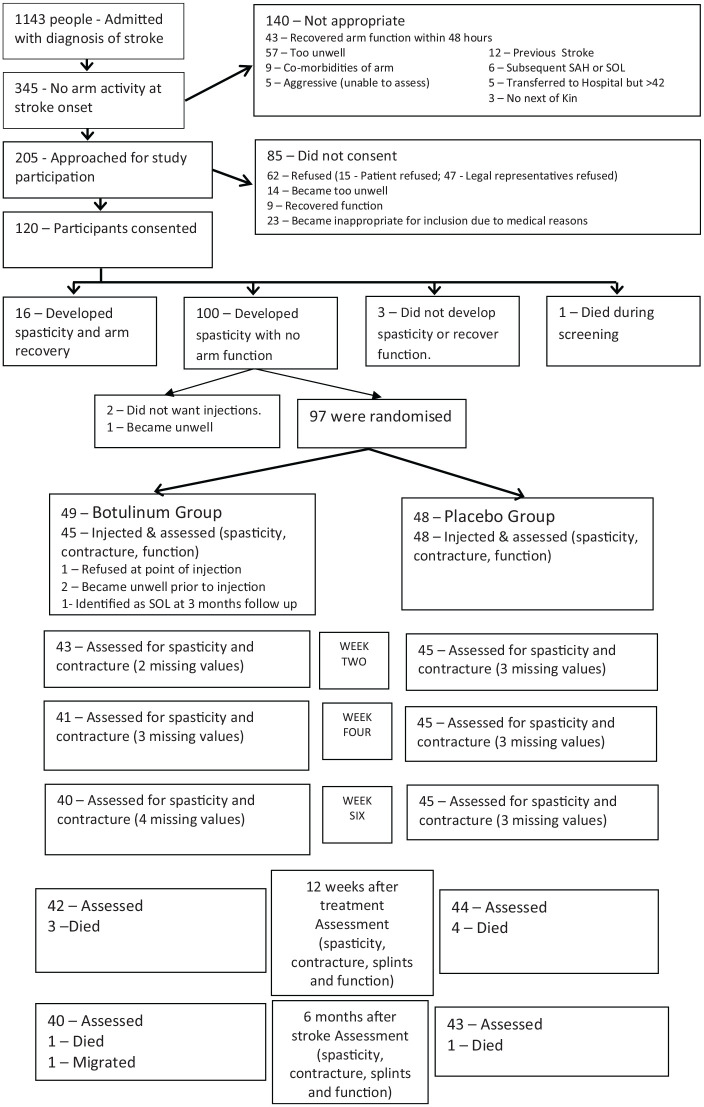
Consort flow chart summarising the flow of participants through the study. The last value was carried forward in case of missing values. SAH: subarachnoid heamorahhage; SOL: space occupying lesion.

Between January 2012 and December 2013, 1143 patients were admitted with stroke, of which 345 had no arm function (95% CI 28% to 33%). One hundred and twenty patients consented to trial participation and one person died during the screening phase leaving a screening sample of 119 patients. (Figure 1)

There was a significant mismatch between the two measures of spasticity. The EMG identified 116 patients as having spasticity in the elbow and wrist respectively. The Tardieu scale identified 61 patients as having spasticity in the elbow and 57 as having spasticity in the wrist. The sensitivity was 0.53 and 0.49 and the elbow and wrist respectively. In the absence of congruence between the two measures of spasticity, a decision was taken to only report the findings from the neurophysiological measure of spasticity, that is, the muscle activity response to a passive stretch.

Of these 119 patients, 97 patients were randomised and 93 were injected (Figure 1) and contributed to the ITT analysis. The treatment and control groups were similar at baseline and the appropriate summary data is presented in Table 2.

**Table 2.** Baseline demographic data for the intention to treat population demonstrating the groups were similar at baseline.

		Treatment group	Placebo group	Total
Number of patients		45	48	93
Age (mean (M) and Standard deviation (SD))		67 (17.1)	68.1 (14.8)	67.5 (15.8)
Sex female (frequency and (%))		21 (47%)	24 (50%)	45 (48%)
Type of Stroke (Frequency)	Infarct (Thrombolysed)	36 (7)	38 (10)	74 (17)
	Haemorrhage	9	10	19
NIHSS total (M(SD))		16 (6.2)	16.4 (6.2)	16.2 (6.2)
NIHSS Sub-Group (M(SD))	Arm	3.6 (0.6)	3.6 (0.6)	3.6 (0.6)
	Leg	2.8 (1.0)	2.9 (1.0)	2.9 (1.0)
	Sensation	1.2 (0.7)	1.2 (0.7)	1.2 (0.7)
	Inattention	1 (0.8)	1 (0.8)	1 (0.8)
Barthel (M(SD))	Pre stroke	19.4 (2.7)	19.5 (1.3)	19.4 (2.1)
	Admission	1.9 (2.9)	1.5 (3.1)	1.7 (3.0)
Action Research Arm Test (M(SD))	Admission	1.0 (2.6)	0.4 (1.7)	0.7 (2.2)
Stroke to Injection (M(SD))	Days	16.8 (8.9)	19.1 (9.5)	18.0 (9.3)

The data from the neurophysiological measurement of spasticity are summarised in Table 3. Immediately following treatment, spasticity in the elbow flexors and wrist flexors decreased in the treatment group. This decrease was seen for six weeks and four weeks in the elbow and wrist of the treatment group respectively. It is important to note, that although the spasticity in the treatment group started increasing, after this early decrease, it was always lower than that measured in the control group. At the elbow, the differences were significant between weeks two and twelve, and, at the wrist the differences were significant between the weeks two and six.

**Table 3.** Spasticity was directly measured using a neurophysiological approach (EMG activity of the flexors during a slow stretch). This table summarises the data from the repeated measurement taken at baseline, 2-weeks, 4-weeks, 6-weeks and 12-weeks post injection, and six months post stroke. The mean and standard deviation for the placebo and treatment group are reported for each time point of measurement. In addition, the mean difference (MD), the 95% Confidence Interval (95% CI) of this difference and the respective *P*-values are reported. Values <0.05 are in bold text.

		Baseline	Week 2	Week 4	Week 6	Week 12	Month 6
Elbow EMG at slow stretch (µV)	Placebo	6.9 (8.3)	10.5 (16.4)	10.8 (12.7)	13.1 (14.4)	14.2 (27.9)	11.6 (16.5)
	Treatment	8.9 (10.9)	3.4 (2.9)	4.1 (3.0)	3.7 (2.7)	5.8 (5.4)	8.1 (9.3)
	MD	2.0	7.1	6.7	9.4	8.5	3.5
	95% CI	−2 to 6	−12 to −2	−11 to −3	−14 to −5	−17 to 0	−9 to 2
	*P* value	0.31	**0.007**	**0.002**	**<0.00** **01**	**0.045**	0.21
Wrist EMG at slow stretch (µV)	Placebo	4.6 (5.8)	8.5 (7.5)	8.1 (6.0)	10.9 (10.6)	7.8 (6.2)	10.1 (7.3)
	Treatment	4 (3.6)	4.1 (3.8)	4.9 (4.7)	5.2 (5.9)	7.5 (7.9)	9.3 (9.2)
	MD	0.6	4.4	3.1	5.8	0.3	0.8
	95% CI	−2.5 to 1.4	−7 to −1.8	−5.6 to −0.7	−9 to −2	−3.3 to 2.7	−4.4 to 2.7
	*P* - value	0.57	**0.001**	**0.01**	**0.002**	0.85	0.58

The data related to elbow contractures (measured as stiffness and passive range of extension) are presented in Table 4. In the control group, the stiffness about the elbow continued to increase until the end of the study period. In the treatment group, there was an immediate and significant decrease in stiffness immediately following treatment. The stiffness then gradually started to increase, however, the stiffness was always lower than that measured in the control group, though not statistically significant. At base line it was possible to achieve anatomical zero in both groups. Over the study period both groups lost passive range of motion and the loss was greater in the control group. The rate in the loss of passive range of extension at the elbow was 2.7 degrees/week slower in the treatment group when compared to the control group.

**Table 4.** Elbow flexion contractures were quantified by measuring the stiffness and the passive range of movement about the joint. This table summarises the data from the repeated measurement taken at baseline, 2-weeks, 4-weeks, 6-weeks and 12-weeks post injection, and six months post stroke. The mean and standard deviation for the placebo and treatment group are reported for each time point of measurement. In addition, the mean difference (MD), the 95% Confidence Interval (95% CI) of this difference and the respective *P*-values are reported. Values <0.05 are in bold text.

ELBOW		Baseline	Week 2	Week 4	Week 6	Week 12	Month 6
Stiffness at slow stretch (mN/deg)	Placebo	12.7 (10.2)	13.5 (10.1)	13.6 (10.0)	14.3 (11.6)	16.8 (15.0)	19.0 (14.7)
	Treatment	14 (11.0)	9.1 (6.7)	10.3 (12.6)	11.7 (8.2)	14.6 (12.6)	15.9 (16.2)
	MD	1.3	4.4	3.3	2.6	2.2	3.1
	95% CI	−3.1 to 5.7	−8 to −0.7	−8 to 2	−7 to 2	−7.5 to 3.6	−9.7 to 3.4
	*P* value	0.55	**0.02**	0.21	0.23	0.48	0.34
Passive range of movement slow stretch (degrees)*	Placebo	−0.4 (3.3)	1.7 (10.0)	3.4 (11.9)	4.4 (11.8)	9.5 (18.9)	9.7 (17.7)
	Treatment	−0.6 (4.5)	0.6 (5.2)	0.7 (5.3)	1.4 (7.5)	2.9 (7.6)	5.0 (11.4)
	MD	0.2	2.2	2.6	3	6.6	4.7
	95% CI	1.43 to −1.8	1.7 to −1.1	1.4 to −6.7	1.2 to −7.2	−0.7 to −12.6	1.4 to −10.8
	*P* value	0.82	0.19	0.18	0.15	**0.028**	0.13

* It is important to note that when interpreting passive range of movement at the elbow a score of zero is indicative of full extension being possible and a score of greater than zero is indicative of a shortening in the flexors.

The data related to wrist contractures (measured as stiffness and passive range of extension) are presented in Table 5. In the control group, the stiffness about the wrist continued to increase until the end of the study period. In the treatment group, there was a decrease in stiffness immediately following treatment and the stiffness remained low until six weeks after treatment. The stiffness then gradually started to increase. Although, the stiffness in the treatment group was always lower than that measured in the control group the differences were not statistically significant. The passive range of extension mirrored the changes in stiffness, that is, the control group showed a systematic loss whilst the treatment group showed a period of plateau until after the week six measurement after which a loss in passive extension was observed. The passive range of extension was higher in the treatment group at all time points after treatment and was only significant at the week six measurement. The rate in the loss of passive range of extension at the wrist was 1.8 degrees/week slower in the treatment group when compared to the control group.

**Table 5.** Wrist flexion contractures were quantified by measuring the stiffness and the passive range of movement about the joint. This table summarises the data from the repeated measurement taken at baseline, 2-weeks, 4-weeks, 6-weeks and 12-weeks post injection, and six months post stroke. The mean and standard deviation for the placebo and treatment group are reported for each time point of measurement. In addition, the mean difference (MD), the 95% Confidence Interval (95% CI) of this difference and the respective *P*-values are reported. Values <0.05 are in bold text.

Wrist		Baseline (BL)	Week 2	Week 4	Week 6	Week 12	Month 6
Stiffness at slow stretch (mN/deg)	Placebo	11.8 (5.5)	13.3 (4.8)	13.8 (6.3)	14.8 (9.1)	15.0 (7.6)	20.0 (12.8)
	Treatment	13.1 (6.3)	11.8 (8.5)	11.6 (7.1)	11.6 (7)	15.4 (10.6)	17.2 (11.5)
	Mean Diff.	1.3	1.6	2.2	3.1	−0.4	2.8
	95% CI	−1.1 to 3.7	−4.6 to 1.3	−5.1 to 0.7	−6.8 to 0.3	−3.4 to 4.3	−7.8 to 2.2
	*P* value	0.29	0.28	0.14	0.07	0.82	0.27
Range of extension at slow stretch (degrees)	Placebo	84.4 (10.3)	74.9 (16.3)	72.4 (15.8)	63.8 (19.5)	58.4 (28.5)	56.4 (37)
	Treatment	82.2 (11.3)	78.5 (14.1)	78.1 (15.9)	75.6 (18.7)	65.4 (28.6)	65.5 (31.4)
	Mean Diff.	2.2	3.6	5.7	11.8	7.1	9.1
	95% CI	−6.7 to 2.2	−2.8 to 10.0	−1.2 to 12.6	3.8 to 19.8	−4.7 to 18.9	−5.1 to 23.3
	*P* - value	0.32	0.27	0.11	**0.004**	0.24	0.2

At three months two patients in the treatment group needed splints when compared to 12 in the placebo group (OR =7.2, 95% CI was 1.5 to 34.1). At six months four patients in the treatment group required a splint compared to the 14 in the placebo group (OR = 4.2, 95% CI was 1.3 to 14.0).

At three months post injection, the mean ARAT score for the control group was 9.5 (SD = 17.95) and for the treatment group this was 11.9 (SD = 19.23), the differences were not significant (mean difference = 2.4, 95% CI was –5.3 to 10.1, *P* = 0.53). At six months post stroke, the mean ARAT score for the control group was 12.4 (SD = 20.7) and for the treatment group this was 15.3 (SD = 21.6), the differences were not significant (mean difference = 2.9, 95% CI was -5.8 to 11.6, *P* = 0.51).

There were no adverse reactions in either group. Six month mortality was similar in both groups: four in the treatment and five in the placebo.

## Discussion

This study has shown that post stroke spasticity occurs significantly earlier than previously reported and has demonstrated that the Tardieu Scale lacks sensitivity in identifying these patients. This inability of commonly used clinical scales to measure spasticity has been previously reported and discussed.^6^

Additional treatment with Botulinum toxin on the first presentation of spasticity (measured neurophysiologically) did lead to the expected reduction in spasticity and the physiological effects of the treatment were consistent with the pharmacokinetics of the drug, that is, effects lasted between four to six weeks.^15^ One hypothesis was that early treatment would lead to a reduction or prevention of contractures and the data suggests that following a single cycle of Botulinum toxin we reduced the rate of contracture formation and also reduced the need for additional treatment with splints.

This study confirms the findings from three previous studies in terms of the short term effects of treatment on spasticity and in the longer term on stiffness. Three previous studies that have specifically investigated Botulinum toxin in the early stage after stroke provided evidence that it may be effective in reducing stiffness.^13,19,20^ One study^13^ used the same technique as our study to identify spasticity whereas the two other studies^19,20^ used variations of the Modified Ashworth Scale to identify and assess stiffness as an indirect measure of spasticity.^21^

The reduction in the rate of contracture formation seen during the first six weeks of the treatment was not sustained. The most plausible explanation for the reduction in rate of contractures in the early stages was related to reduced levels of flexor muscle spasticity. Once the effects of Botulinum toxin on the motor neurone junction were reversed, the risks of the joints being held in flexed position would have increased, particularly if the patients had not recovered useful arm function. This could explain the reversal in the rate of contracture formations after the six weeks window.

One could therefore hypothesise that, for patients who have not recovered useful function, additional treatments or larger initial doses of toxin may be required. Increasing the doses could lead to a more diffuse weakening effect which would potentially be detrimental to recovery. Treatment is normally repeated every three months and one may need to consider repeating treatment every two months as the clinical befit does not last three months. Further research is required to investigate these two options in stroke. A third option is to consider a range of concomitant treatments that might include splinting in addition to cyclical electrical stimulation and botulinum toxin.

The response to treatment and contracture development was different between the elbow and the wrist with a greater treatment effect observed at the elbow. One explanation is that there is a greater proportion of connective tissue present in the forearm flexors may lead to a greater amount of collagen crosslinks which is a major cause of stiffness.^22^ Despite use of EMG/e-stim or ultrasound localisation techniques there is the possibility the treatment was better targeted for the muscles that control elbow flexion as opposed to the muscles that control wrist and finger flexion.

The second hypothesis was that using botulinum toxin to reduce spasticity and contractures could lead to an improvement in arm function. There were two plausible pathways to facilitate the recovery of arm function. The first was that, unlike systemic antispastic agents, botulinum toxin will not depress the central nervous system and therefore not interfere with the potential for neuronal recovery.^16–18^ The second was that the short term prevention of contractures and the suppression of spasticity, whilst simultaneously providing treatment with electrical stimulation, would improve the recover the recovery potential in these severely disabled patients. The results in study suggest that early treatment with botulinum toxin did not facilitate the recovery of arm function in the short term. The most likely reason for a failure to demonstrate functional benefit was that many of our patients may have had severe strokes with a lower potential for recovery^24^ and a paucity in the access to an appropriate intensity/dose of rehabilitation for the upper limb.^25^ It was, however, reassuring that the early treatment of spasticity with botulinum toxin appears not to have interfered with the recovery of arm function in those who are likely to have had the potential for functional recovery.

### Strengths and limitations

It is possible that the treatment with electrical stimulation may have contributed to recovery by increasing the cortical plasticity potential through increased excitability in the sensory-motor cortex.^23^ The impact of this confounder was limited as patients in both groups received treatment with electrical stimulation. Future studies may want to delineate any interaction or confounding effects between these two treatments in relation to contracture development and functional improvement. A further issue was that the original aim was to recruit patients in the early stages after a stroke, that is, before spasticity developed. As a result of delays in getting consent from consultees some patients had already developed spasticity on day of recruitment and this could have reduced the impact of treatment.

On the basis of this study there is justification for a larger multi-centre clinical trial (with stratification/minimisation based on predicted recovery potential) with health economic analysis to investigate the potential efficacy of this treatment further. Further mechanistic studies aimed at more effectively targeting the treatment, the dose of treatment and the repetition cycles for the treatment are also required as treatment selection was based on consensus guidelines as opposed to research evidence.

## Conclusion

This study has demonstrated that spasticity can be identified early after stroke and that the current clinical scales lack the sensitivity to identify the early signs of spasticity. Treating spasticity with Botulinum toxin reduces spasticity and the rate of contracture formation. The effects of treatment were consistent with the pharmacokinetic behaviour of the Botulinum toxin, long terms clinical benefits, such as reducing the need for contracture management devices is possible. Early treatment with Botulinum toxin did not facilitate recovery of arm function, however, there was no evidence that treatment hindered functional recovery.

**Clinical message**

- Early use of Botulinum toxin, in patients who have spasticity, is safe following stroke and is not detrimental to functional recovery.
- A single cycle of Botulinum toxin reduces the rate of contracture formation and reduced the need for concomitant contracture treatment such as splinting.
- Botulinum toxin does not improve function in people with severe stroke who have developed spasticity.

## Supplemental Material

Appendix_1 – Supplemental material for Can the early use of botulinum toxin in post stroke spasticity reduce contracture development? A randomised controlled trial Click here for additional data file. Supplemental material, Appendix_1 for Can the early use of botulinum toxin in post stroke spasticity reduce contracture development? A randomised controlled trial by Cameron Lindsay, Sissi Ispoglou, Brinton Helliwell, Dawn Hicklin, Steve Sturman and Anand Pandyan in Clinical Rehabilitation
