# Supplementary material for: Can the early use of botulinum toxin in post stroke spasticity reduce contracture development? A randomised controlled trial
Source: Clin Rehabil. 2020 Oct 11;35(3):399–409. doi: 10.1177/0269215520963855 (PMC7944432; doi:10.1177/0269215520963855)
Supplement: Appendix_1 – Supplemental material for Can the early use of botulinum toxin in post stroke spasticity reduce contracture development? A randomised controlled trial [file Appendix_1.pdf]

Appendix 1: A brief description of the method used to directly measure spasticity.

Data for spasticity, stiffness, and passive range of movement were all measured in the same procedure which has been described previously.[9,10,12,13] In a sitting or half lying position a research therapist moved the joint from full flexion to full extension, using two manually controlled velocities (i.e. first with a low velocity and then with a high velocity). The fingers were maximally extended during wrist extension measurement and joints were extended to perception of end range by the research therapist (or to pain onset if this was earlier).

To measure spasticity, muscle activity data was collected using bipolar surface EMG active electrodes (SX230) (Biometrics Ltd, UK.) placed over biceps brachii or flexor carpi ulnaris. To measure contractures, angular data was collected with a flexible electrogoniometer (SG 110). Force data was collected with a Myometer (M550) (Biometrics Ltd, UK). These transducers were sampled at 1000Hz using a MWX8 DataLOG (Biometrics Ltd, UK.) and stored for post-hoc analysis. The outcome measures were later extracted from the data files using a customised programme (Mathcad 15, PTC, USA.)

Spasticity was quantified as the mean muscle activity of the flexors throughout the movement. The mean muscle activities during low velocity movements are presented. Contractures were quantified as loss of passive range of movement measured by the maximum angle of joint extension achieved during a slow passive stretch and resistance to the passive movement measured as the slope of force over angle curve using a least square regression method.[12]
